# Management of Dental Extraction in Liver Cirrhosis: A Case Report

**DOI:** 10.7759/cureus.80388

**Published:** 2025-03-11

**Authors:** Ibtihag S Elnaem, Ebtehal M Aloudah, Hawaa A Essa, Layla H Alenzi, Albandri M Alghris

**Affiliations:** 1 Oral and Maxilofacial Surgery, College of Dentistry, University of Hail, Hail, SAU; 2 General Medicine, University of Hail, Hail, SAU; 3 Dentistry, Fajr College for Science and Technology, Khartoum, SDN; 4 Dentistry, College of Dentistry, University of Hail, Hail, SAU

**Keywords:** hail, liver cirrhosis, omfs, oral and maxillofacial surgery, saudi arabia

## Abstract

Cirrhosis prevalence varies worldwide, with high incidence rates observed in populations with high rates of hepatitis infection and alcohol consumption. Portal hypertension, ascites, hepatic encephalopathy, and heightened vulnerability to infections are common in patients with advanced liver disease. In addition to impairing hemostasis, immune function, and metabolism, chronic liver disease increases susceptibility to oral and systemic infections. Because it affects infection risk, drug metabolism, and hemostasis, liver illness and dentistry interact in a crucial way. Patients with liver illness are more susceptible to oral infections, and impaired liver function can change how well regularly given drugs are cleared. A 54-year-old female with liver cirrhosis and a significant medical history presented with pain in the lower left teeth. This case study emphasizes the challenges involved in managing patients with liver cirrhosis and preventing postoperative complications using the essential examination for accurate and effective treatment in an academic-based clinic.

## Introduction

Liver disease encompasses a wide range of pathological conditions that impair hepatic function, including viral hepatitis (e.g., hepatitis B and C), alcoholic liver disease (ALD), non-alcoholic fatty liver disease (NAFLD), cirrhosis, and hepatocellular carcinoma [1]. As the largest solid organ in the body, the liver plays a fundamental role in metabolic regulation, detoxification, protein synthesis, and immune modulation. When liver function is compromised, it can lead to severe systemic complications, including coagulation abnormalities, metabolic imbalances, and multi-organ dysfunction [2]. Liver diseases are categorized based on their etiology, progression, and severity. Acute liver failure (ALF) presents with sudden hepatic dysfunction, whereas chronic liver disease (CLD) develops gradually over time, often progressing to cirrhosis and end-stage liver disease. Patients with advanced liver disease frequently experience portal hypertension, ascites, hepatic encephalopathy, and increased susceptibility to infections due to impaired immune function [3]. The systemic consequences of liver disease extend beyond hepatic dysfunction, affecting hemostasis, immunity, and metabolism. One of the most significant complications is coagulation disorder, as the liver is responsible for synthesizing clotting factors. Patients with cirrhosis often develop thrombocytopenia and prolonged prothrombin time, increasing their risk of excessive bleeding during surgical and dental procedures [4]. Additionally, hepatic encephalopathy, a neuropsychiatric syndrome resulting from the accumulation of ammonia and other toxins, can impair cognitive function, leading to difficulties in maintaining proper oral hygiene and adhering to dental care instructions [5,6].

Patients with CLD also exhibit immune dysfunction, making them more vulnerable to systemic and oral infections [7]. Hepatic dysfunction alters the production of immune mediators, leading to impaired neutrophil function and a higher risk of bacterial and fungal infections, including periodontitis and oral candidiasis. Furthermore, metabolic complications, such as insulin resistance in NAFLD and electrolyte imbalances in cirrhotic patients, can further impact overall health and influence dental treatment considerations [8]. 

The interplay between liver disease and dentistry is critical due to the impact on hemostasis, drug metabolism, and infection risk [9]. Before performing invasive dental procedures, clinicians must assess the patient’s coagulation status, including platelet count, prothrombin time (PT), and international normalized ratio (INR) [10,11]. If the bleeding risk is high, consultation with a hepatologist may be necessary to optimize hemostasis through platelet transfusions or vitamin K administration [12]. 

Another major concern is drug metabolism [13]. The liver is the primary site for drug biotransformation, and impaired hepatic function can alter the clearance of commonly prescribed medications such as local anesthetics, antibiotics, and analgesics. Acetaminophen, for instance, is hepatotoxic at high doses and should be used cautiously in patients with liver disease. Similarly, nonsteroidal anti-inflammatory drugs (NSAIDs) should be avoided due to their potential to exacerbate gastrointestinal bleeding in cirrhotic patients [14].

Patients with liver disease are also at higher risk for oral infections, including periodontitis and osteomyelitis, as well as poor wound healing due to malnutrition [15]. Immune dysfunction necessitates meticulous postoperative care, and prophylactic antibiotics may be required for immunocompromised individuals [16]. Additionally, liver disease patients on bisphosphonates for osteoporosis management are at risk for medication-related osteonecrosis of the jaw (MRONJ), which requires careful surgical planning and preventive strategies [17]. The study intends to evaluate how liver disease affects dental treatment, taking into account possible side effects such as immunological dysfunction and coagulation abnormalities, and to develop safe dental procedures.

## Case presentation

**Figure 1:**
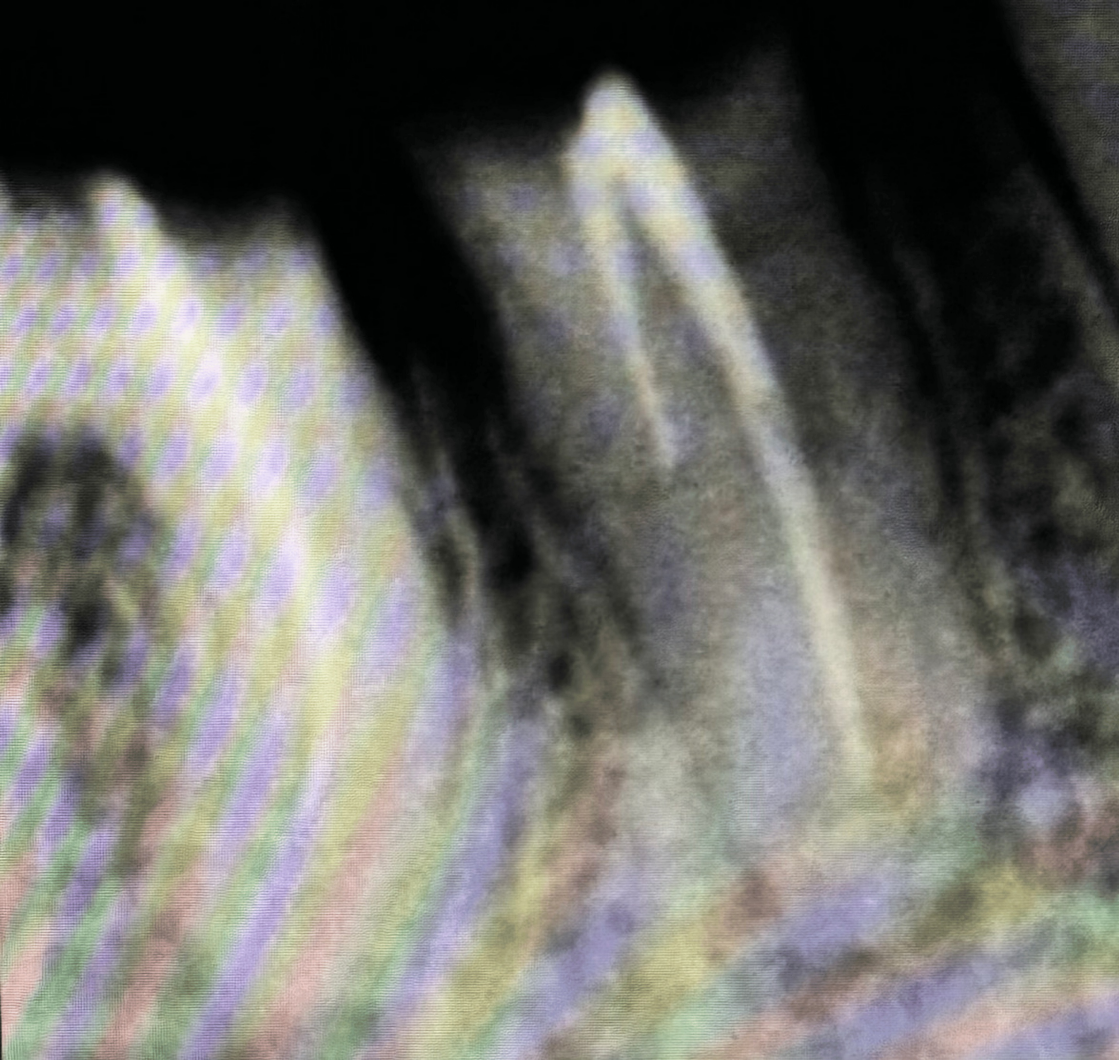
Periapical X-ray of #45

**Figure 2:**
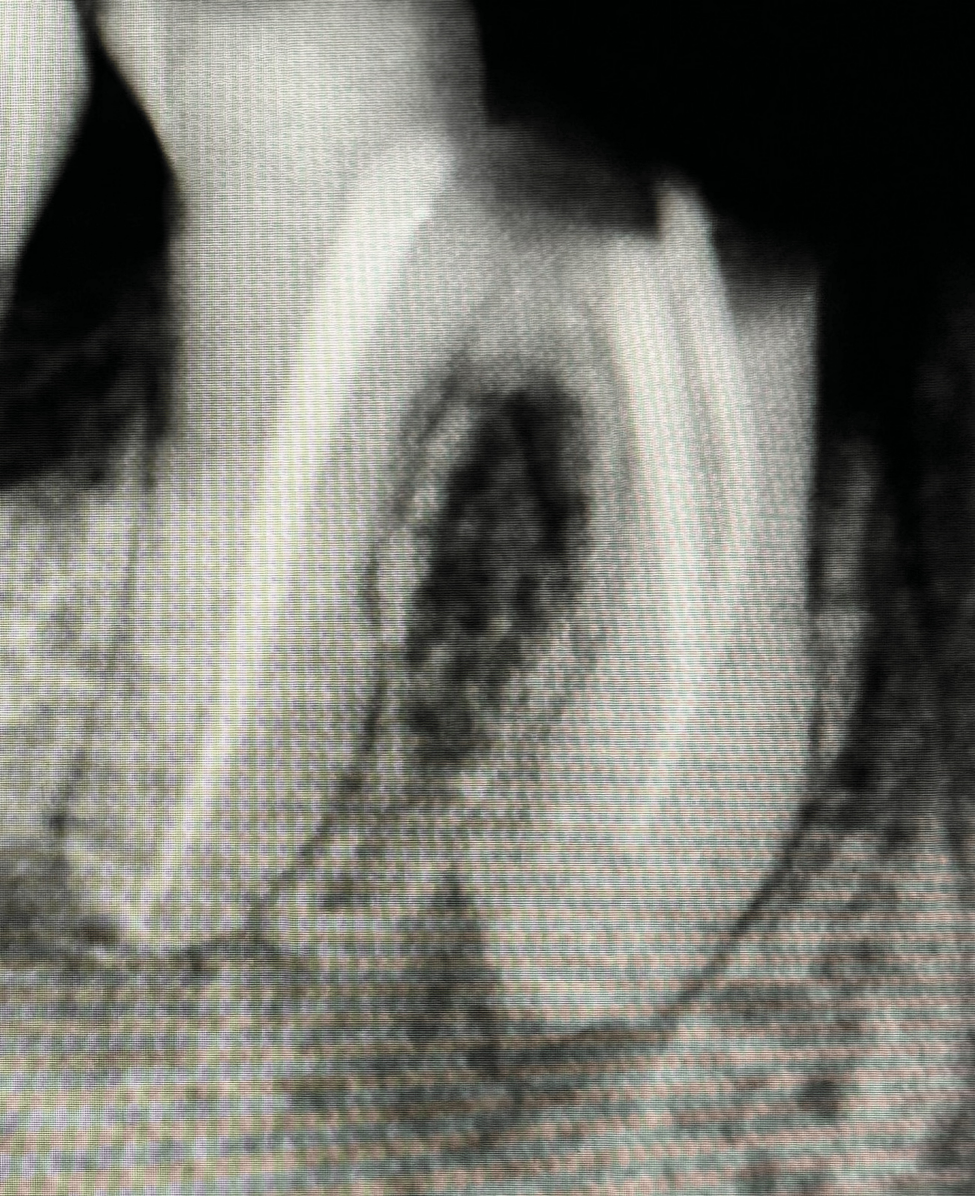
Periapical X-ray of #46

A 54-year-old female with liver cirrhosis presented to the Department of Oral and Maxillofacial Surgery at the University of Hail Dental Polyclinics with a chief complaint of pain. Despite the underlying liver disease, the patient's overall health remained stable, with no acute systemic signs or symptoms. Clinical examination revealed a destructive tooth in #46 and a remaining root in #45. The patient has good oral hygiene with a history of previous dental extraction but a multiplate treatment with the endodontic and restorative department. Following an initial assessment, the patient underwent a Periapical X-ray for #45 and #46 (Figures 1, 2).

Preapical radiograph of tooth #46 showing a previous endodontic treatment. Both teeth #45 and #46 are indicated for extraction.

Medical history

The patient has a medical history of liver cirrhosis associated with moderate ascites. The patient has a recent history of weight loss and fatigue. The patient is currently on carvedilol, furosemides, ursodeoxycholic acid, dietary supplements (calcium, magnesium, vitamin C, zinc, and lecithin), and oral lactulose. No known drug allergies. The patient undergoes endoscopic variceal ligation every four months as a treatment for esophageal varices. There is no family history of liver disease; however, there is a family history of diabetes.

Clinical finding 

Physical Examination

The patient weighs 80 kg and is 170 cm tall. From her external appearance, the patient appears well-groomed, well-fed, and free of anemia and jaundice. The patient shows finger clopping, an indication of a liver disorder.

Extraoral Examination

There is no facial asymmetry or swelling in the patient. There are no lumps or painful spots when the lymph nodes are palpated, indicating the absence of lymphadenopathy. There should be no discomfort in any of the temporomandibular joints or masticatory muscles, nor any restricted openings, deviations, or asymmetries.

Intraoral Examinations

Upon examination, palpation, and percussion, teeth typically exhibit arrested caries, a defective restoration, no gingival recession, and mobility. However, based on the patient's chief complaint, the lower left jaw is being severed, revealing a remaining root in #45 and a destructive tooth in #46.

Investigation 

Radiographic Investigation

Periapical X-ray revealed that both #45 and #46 had prior endodontic treatment. The rationale for extraction is increased by severe bone loss and furcation involvement, as shown previously in Figures 1, 2.

Laboratory Investigation

Laboratory showed an INR of 1.29 and a count of 86,000/uL platelet that suggests mild to moderate thrombocytopenia. The albumin was 2.9 g/dL, and Bilirubin was 1.4 mg/dL (Table 1).

**Table 1:** Laboratory results CBC: complete blood count; RBC: red blood cells; HgB: hemoglobin; HCT: hematocrit test; MCV: mean corpuscular volume; MCH: mean corpuscular hemoglobin; MCHC: mean corpuscular hemoglobin constriction; WBC: white blood cells; LFT: liver function test; PT: prothrombin time; INR: international normalized ratio; RFT: renal function tests

Parameters	Results	Normal range
CBC		
RBC	3.82x10^6^/uL	4-5.5
HgB	12.5 g/dL	12-16
HCT	34.7%	33-51
MCV	90.8 fL	76-98
MCH	32.7 pg	26-34
MCHC	36%	30-36
WBCs	4.98x10^3^/uL	4-11
Neutrophils percentage test	63.5%	40-70
Lymphocyte percentage test	23.5%	20-44
Monocytes percentage test	9.2%	2-9
Eosinophils percentage test	3.2%	1-6
Neutrophils/uL	3.16x10^3^/uL	2-7
Lymphocytes/uL	1.17x10^3^/uL	1-3.5
Monocytes/uL	0.46x10^3^/uL	0.2-1
Eosinophils/uL	0.16x10^3^/uL	0.2-01
Basophils/uL	0.03x10^3^/uL	0.02-0.1
Platelets	86x10^3^/uL	150-450
LFTs		
Albumin	2.9 g/dL	3.50-5.20
Bilirubin	3.2 mg/dL	0.00-1.0
PT	17.7 sec	10.0-15.0
INR	1.29	0.90-1.20
RFTs		
Creatinine	0.6 mg/dL	0.60-1.0

Management

The patient receives an overall score of eight points, placing them in class B according to the Child-Pugh criteria for evaluating the severity of liver disease. Excessive bleeding during and after tooth extraction is a risk for the patient. Vital signs were checked; oxygen saturation was 97%, and blood pressure was 125/80. After administering a local anesthesia injection, the patient is instructed to rinse her mouth for one minute with 15 mL of chlorhexidine as mouthwash.

To reduce systemic effects, lidocaine (xylocaine) 2% and epinephrine 1:100,000 were used for local anesthesia. The inferior alveolar nerve block technique was used to deliver a single carpule of local anesthesia. 

**Figure 3:**
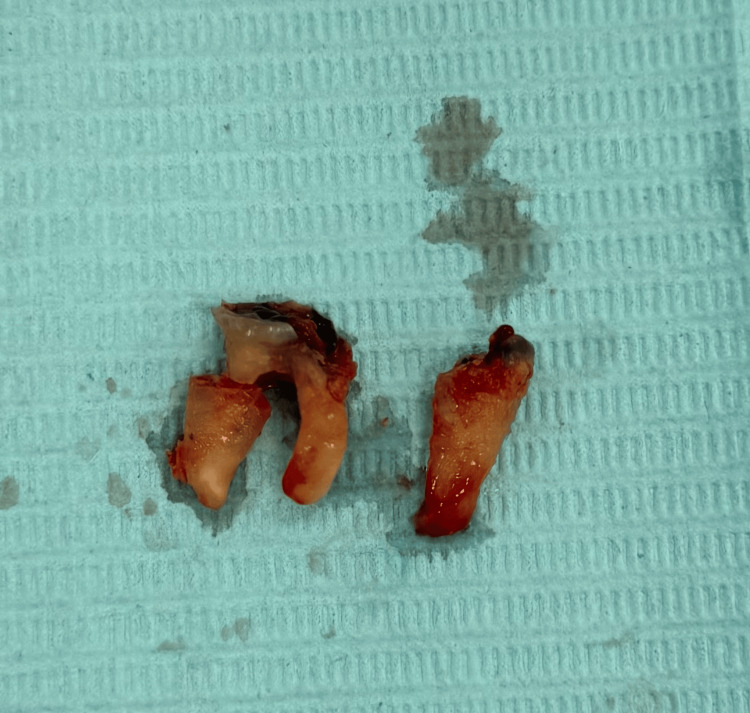
After removal of #45 and #46

Lower molar forceps, mesial and distal Cryer's elevators, and Coupland's chisel elevators were chosen as the procedure's tools. The remaining roots of #45 and #46 were removed effectively, as shown in Figure 3.

One of the issues in such circumstances causes bleeding, which was managed with gel foam and a simple interrupted suture. Following surgery, the patient was monitored for one hour to ensure that the bleeding was under control, and they were prescribed 500 mg of acetaminophen as needed and 650 mg of augmentin every eight hours for seven days.

## Discussion

This case highlights the challenges associated with performing dental extractions in patients with liver cirrhosis, particularly regarding hemostasis, infection control, and pharmacological management. Given the patient’s classification as Child-Pugh Class B and laboratory findings indicating thrombocytopenia (86,000/μL) and a slightly elevated INR (1.29), the risk of excessive bleeding was a primary concern. As observed in previous studies, patients with liver cirrhosis and an INR ≤2.5, along with platelet counts above 30,000/μL, have a reduced risk of severe postoperative bleeding, often allowing for safe minor surgical interventions without the need for blood transfusions (Perdigão et al., 2012) [18]. However, given the variability in hemostatic function among cirrhotic patients, the decision to implement local hemostatic agents and prolonged monitoring was essential in preventing complications.

The use of chlorhexidine mouthwash preoperatively was a key infection control measure. Liver cirrhosis is associated with an altered immune response, increasing susceptibility to systemic and oral infections, such as periodontitis and osteomyelitis. Chlorhexidine, as a broad-spectrum antiseptic, has been demonstrated to significantly reduce bacterial load and lower the risk of postoperative infections, which is particularly beneficial in immunocompromised patients. Furthermore, the prescription of Augmentin (amoxicillin/clavulanic acid) was justified by the patient’s increased vulnerability to infections, as hepatic dysfunction can impair the body’s ability to clear bacterial pathogens effectively.

Pharmacological considerations were particularly important in this case. The choice of 2% lidocaine with 1:100,000 epinephrine for local anesthesia was guided by the need to minimize systemic absorption and avoid hemodynamic instability. While some literature suggests that epinephrine should be used cautiously in cirrhotic patients due to its potential vasoconstrictive effects, it remains a preferred choice when administered in controlled doses to prolong anesthesia and reduce intraoperative bleeding [19]. Postoperatively, acetaminophen was chosen as the analgesic agent due to its relative safety in patients with liver disease when administered within therapeutic limits. NSAIDs were avoided due to their hepatotoxic potential and their role in exacerbating gastrointestinal bleeding, a common complication in cirrhotic patients.

Radiographic findings played a crucial role in treatment planning. The periapical radiograph revealed previous endodontic treatment in #46, along with severe bone loss and furcation involvement in both #45 and #46. These findings confirmed the necessity of extraction due to the compromised structural integrity of the teeth and the likelihood of future complications if left untreated. While root canal retreatment may have been considered under different circumstances, the risk of persistent infection and the patient’s overall systemic condition justified extraction as the most appropriate intervention.

When compared to similar cases in the literature, Patel et al. (2016) reported the management of a cirrhotic patient with a significantly lower platelet count (32,000/μL) who required multiple extractions. In contrast to our case, their approach involved preoperative platelet transfusions and adjunctive hemostatic agents such as tranexamic acid and fibrin sealants [20]. While our patient did not require transfusions, successful hemostasis was achieved using local measures alone, aligning with Perdigão et al.’s findings that minor oral surgeries can be safely performed in cirrhotic patients with moderate thrombocytopenia [18]. These comparative findings highlight the necessity for individualized bleeding control strategies tailored to each patient’s specific coagulation status.

## Conclusions

This case highlights the difficulties in treating tooth extractions in patients with liver cirrhosis, with an emphasis on pharmaceutical therapy, infection control, and hemostasis. Due to the patient's Child-Pugh Class B status, thrombocytopenia, and slightly raised INR, it was necessary to take precautions to reduce the risk of bleeding. Patient safety during the procedure was ensured in large part by the use of local hemostatic medications and close observation.
